# *In vitro* investigation of elevated hemolysis susceptibility in neonatal blood

**DOI:** 10.3389/fped.2025.1616084

**Published:** 2025-08-26

**Authors:** Jan Heyer, Stella Volmering, Camila Hoyos-Banchon, Sarah Koc, Lydia Jeremiah, Ulrich Steinseifer, Thorsten Orlikowsky, Sebastian V. Jansen, Johanna C. Clauser, Mark Schoberer

**Affiliations:** ^1^ Department of Cardiovascular Engineering, Institute of Applied Medical Engineering, Medical Faculty, RWTH Aachen University, Aachen, Germany; ^2^ Department of Pediatric and Adolescent Medicine, Neonatology, Medical Faculty, University Hospital RWTH Aachen, Aachen, Germany

**Keywords:** preterm infants, artificial placenta, neonatal blood hemolysis, neonatal ECMO, porcine vs. neonatal blood

## Abstract

**Introduction:**

Hemolysis is a relevant complication and is responsible for morbidity and mortality of neonatal extracorporeal membrane oxygenation (ECMO) therapy. For novel therapies like artificial placenta, hemolysis could also lead to complications or therapy failure, especially since the targeted patients are born at the borderline of viability. Standardized *in vitro* blood testing using animal blood is commonly used to assess the hemolytic potential of newly developed systems during their design and development. However, neonatal human blood differs from animal blood. For example, neonatal blood exhibits a higher erythrocyte volume, lower overall viscosity, and greater erythrocyte elasticity. This study investigates whether the porcine blood analog, commonly used in standardized protocols, can also be used to assess hemolysis in neonatal blood.

**Methods:**

Human neonatal blood was harvested from placentas and umbilical cords of neonates born by cesarean section. Porcine blood was obtained from a local abattoir. Both collection processes followed predefined standardized protocols. Normalized Index for hemolysis (NIH) was calculated based on determined free plasma hemoglobin.

**Result:**

There was a significantly (*p* < 0.05) higher normalized index of hemolysis in the human neonatal blood group (NIH=0.165 g 100 L^−1^; SD=0.082) compared to the porcine group (NIH=0.101 g 100l^−1^; SD=0.038). In contrast, under static reference conditions, neonatal blood exhibited lower hemolysis (NIH=0.025 g 100 L^−1^; SD=0.018) than porcine blood (NIH=0.055 g 100l^−1^; SD=0.038).

**Discussion:**

In standardized *in vitro* hemolysis testing, porcine blood might not serve as a suitable analog for human neonatal blood, as it significantly underestimates the hemolysis potential observed in neonatal blood.

## Graphical Abstract

**Figure:**
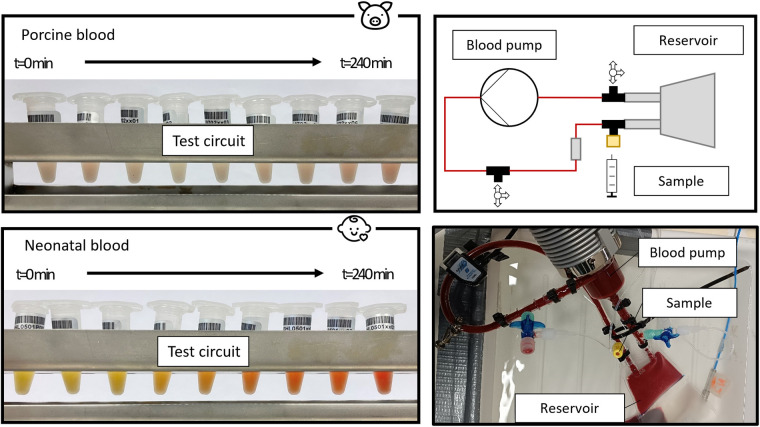

## 1 Introduction

Every year, approximately 13 million children are born preterm worldwide. Among these, an estimated 900,000 cases (6.7%) result in significant morbidity (1). The availability and quality of neonatal intensive care units (NICUs) play a critical role in deciding the outcomes for the smallest group of patients: extremely preterm infants (EPIs) born at or before 28 weeks of gestation. Apart from medicines, medical technology plays a crucial role in NICU care. It enables vital parameter monitoring, supports various modes of mechanical ventilation, and provides incubators that maintain an air-based microenvironment to preserve body temperature and prevent infections. The continuous development of these therapeutic modalities has allowed infants born as early as 22 weeks of gestational age (GA) to survive in the most advanced settings (2). However, the burden of morbidity and mortality among extremely preterm infants remains high. In a cohort of 10,877 EPIs born between 2013 and 2018 in 19 academic US tertiary care perinatal centers, Bell et al. reported a survival-to-discharge ratio of 78%, with severe neurological impairment seen in 21% of cases (3). For supporting the most immature infants at the borderline of viability, several research groups worldwide are developing artificial placenta technology (APT). APT can be used as a lung assist device for rescue therapy (4) or as a platform for complete extracorporeal gas exchange, allowing the lungs to remain in a functionless state while continuing to mature. This can be performed either in a state in which only the lungs are isolated from the gas atmosphere using an endotracheal tube (5) or in a completely artificial womb environment (6–8).

**Figure 1:**
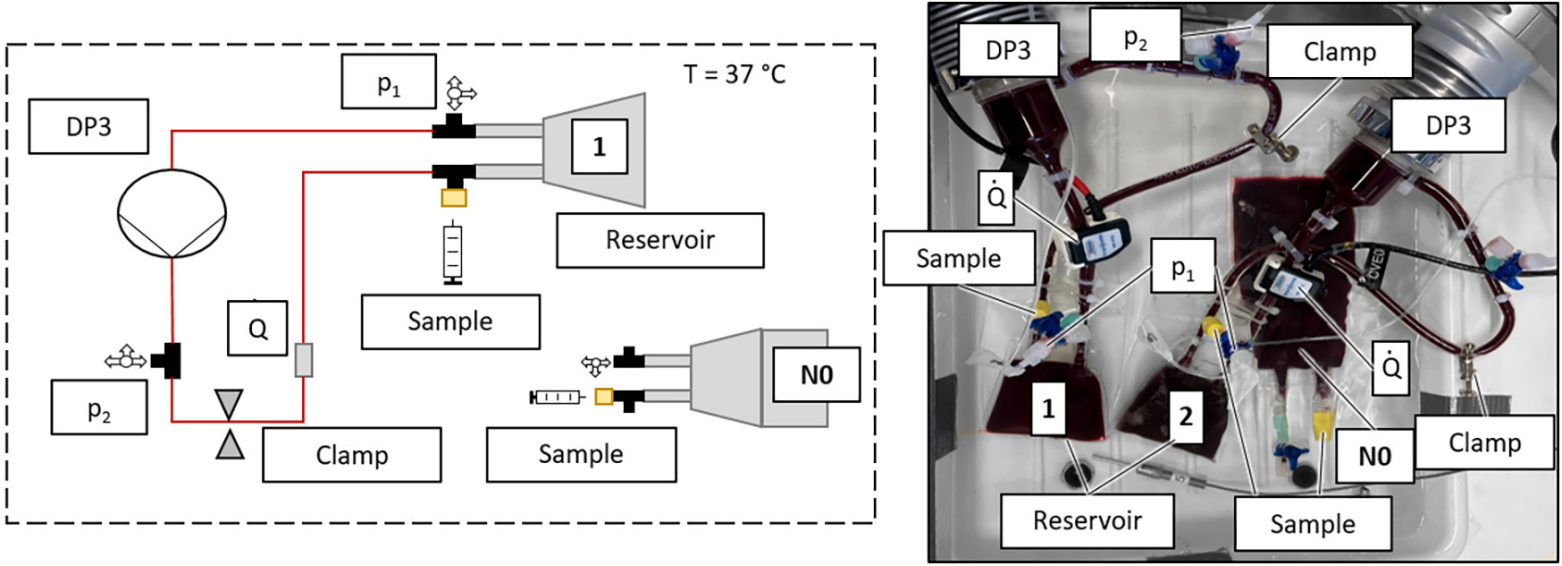
Setup of the *in vitro* hemolysis test circuit (1, 2) with static reference N0: schematic sketch of one test circuit with static reference (left); picture of two test circuits and one static reference (right).

Common to APT is, that the exchange of respiratory gases is maintained via cannulas connected to an extracorporeal membrane oxygenation (ECMO) circuit, driven by the heart of the neonate (6, 9) or an additional external blood pump (10). These technologies aim to resolve existing treatment limitations and establish a bridge-to-life approach.

One of the leading causes of failure in neonatal ECMO, used in infants with a GA > 36 weeks, is hemolysis (11–13). Because of the integral function of ECMO, this problem must also be anticipated in APT. Extracorporeal system development includes standardized *in vitro* testing for hemolysis, which is performed using validated animal blood models, of which the porcine model is most commonly used due to its biosimilarity to adult human blood (21). Although some APT concepts are pumpless, blood pumps are required for *in vitro* hemolysis testing in dynamic circuits for relative comparisons. According to standards (e.g., ASTM 1843), overall hemolysis caused by blood pumps is assessed preclinically using *in vitro* porcine blood models (14, 15). These *in vitro* models were developed based on existing knowledge of the correlations between adult human and porcine blood (14, 16, 17). However, the cell structure and cell counts of neonatal blood differ significantly; e.g., neonatal red blood cells have a nucleus and a different intracellular volume compared to porcine blood (18–21). Furthermore, differences in the composition of neonatal blood, e.g., hematocrit, fibrinogen fraction, and overall cell structure, lead to different viscosity profiles under given shear rates (18, 22, 23). These differences in blood properties raise concerns about the different hemolytic behavior of neonatal blood and the suitability of existing *in vitro* porcine test standards for assessing hemolysis.

This work investigates the differences in hemolysis between neonatal human and porcine blood and evaluates the suitability of porcine blood as an analog fluid for *in vitro* neonatal blood testing. A comparative study was performed using a test circuit with minimal priming volume and standardized hemolysis tests.

## 2 Methods

Human neonatal blood samples were acquired by harvesting umbilical cords along with the placenta from the centralized biomaterial bank (cBMB) of RWTH Aachen University immediately after delivery via cesarean section. The medical procedure was not influenced by the harvesting process. Blood was extracted from the placenta and umbilical vessels only after complete separation of these tissues from both mother and newborn. Written maternal consent was obtained in all cases. The procedure was approved by the ethics committee of the medical faculty of RWTH Aachen University (EK 206/09). Data from the mother, the neonate, and the umbilical cord were pseudonymized using a sample code provided by the cBMB. Access to primary patient data was restricted to the project physicians and the cBMB.

### 2.1 Harvesting of neonatal and porcine blood

Neonatal blood was collected from the umbilical cord and placenta, which were harvested during cesarean sections at the University Hospital, RWTH Aachen University. Immediately after delivery, the placenta and umbilical cord were transferred to a workbench. At the workbench, the clamped umbilical cord was separated from the placenta. The blood from the placenta was collected by hanging and draining the ends of the umbilical vessels into a sampling tube. The blood from the severed umbilical cord was gently pressed out of the cord by hand into a sampling tube. After harvesting, the blood was immediately heparinized with 15,000 IU L^−1^ of high molecular weight heparin (Ratiopharm, Germany). The average harvested amount of blood with this method was preliminarily defined as 45 ml ± 5 ml. The placental and umbilical cord blood was either pooled or used separately in different circuits.

The porcine blood was harvested from a local slaughterhouse. It was immediately treated with 15,000 IU L^−1^ of high molecular weight heparin (Ratiopharm, Germany), 1.6 ml L^−1^ of gentamicin (Ratiopharm, Germany), 1.8 ml L^−1^ of 50% glucose (B.Braun, Germany), and 100 ml L^−1^ of 0.9% saline solution (B.Braun, Germany) after harvesting.

### 2.2 Definition of the population of the donors and experimental exclusion criteria

The minimum number of neonatal blood donors was set to seven during a 6-month harvesting time. By assuming a normal distribution with infinite basic population a confidence interval of 80%, a maximum error of 25% and a conservative estimation of the standard deviation of 50%, seven experiments are required as a minimal sample size. No upper limit was set, and each possible experiment was realized during this time period. Depending on the amount of harvested neonatal blood, one or two parallel test circuits and one static reference were realized. In case of two test circuits, the results of both circuits were summarized as a mean value. The decision process for the number of circuits based on the diluted blood volume is illustrated in Supplementary Material 1.

The same number of experiments from individual donors were performed with porcine and neonatal blood. The porcine blood was always tested using two test circuits and one static reference. For all experiments with two test circuits, the results for each individual are given as the mean of both measurements.

### 2.3 Test setup for hemolysis testing

The standard test circuit for blood pump hemolysis testing according to ASTM 1841-19 (14) was further miniaturized based on the work of Woelke et al. (24, 25) to a priming volume of 55 ml. This was the minimal volume that allowed the implementation of all required devices and components, such as a pump (Delta stream 3, Medos, Stadt, Germany), connectors for sampling and measuring points, and a reservoir (modified blood bag with 20 ml priming volume, Qosina Ronkokoma, USA) without a suction event. A Hoffmann clamp was used to regulate the flow rate. Samples were taken via a port (IN-Stopper, B.Braun Melsungen AG, Germany) using a syringe with cannula (100 Sterican 23G × 1 ¼, B.Braun Melsungen AG, Germany) to ensure the smallest possible sample volume. The complete circuit was placed in a heated water bath to maintain a blood temperature of 37°C. Pressure, flow, and water bath temperature were measured constantly. The test setup is shown in Figure 1. A blood bag filled with blood from the same batch as the test circuit was placed in the water bath as a static reference (N0). Samples were drawn from the static reference as well as from the test circuit(s).

### 2.4 The protocol of hemolysis testing

Deviating from the ASTM standard, the testing time was reduced from 360 min (14), to 240 min due to handling reasons to prevent extensive hemolysis exceeding measurement capabilities (26). The hematocrit (Hct) was adjusted by dilution with 0.9 NaCL solution (B.Braun, Germany) to 30% ± 1%. The blood volume flow target was set to 70 ml min^−1^, which is the flow within an umbilical vein of a fetus at 24 weeks of GA (27). The circuit was primed with the Hct adjusted blood. Proper mixing and heating of the used blood was ensured by slowly increasing the blood volume flow from 10 ml min^−1^ to the 70 ml target flow, with 10 ml min^−1^ increments every 10 min, during the first 60 test minutes. This phase is inspired by the startup phase in neonatal ECMO, whereby the volume flow is increased over a more extended period in a startup phase to accustom the patient to the ECMO (28). After reaching the target flow, the circuit's pressure drop was adjusted to 110 mmHg ± 10 mmHg with the Hoffmann clamp. By choosing a pressure loss above the blood pump of 100 mmHg, the circuit operates within a low-flow, high-pressure area, a worst-case scenario for hemolysis within blood pumps (29). Samples of 1 ml were taken at 0 min, 60 min, 90 min, 120 min and 240 min in each circuit, with 0 min being set, when the target flow was reached. The Hct was determined using density gradient centrifugation. Each sample was used for a full blood count and determination of free plasma hemoglobin.

The free plasma hemoglobin concentration was determined using the cyanohemoglobin method standardized by DIN 58931 (30). Zander et al. (31) showed that the method is also suitable for fetal hemoglobin with a deviation smaller than 1% compared to adult blood. The normalized index of hemolysis (NIH) was calculated for each blood type, based on the measured parameters starting at 60 min after reaching the targeted volume flow until the last sample at 240 min. The equation of the NIH is shown in Equation 1.

(1) $$\mathrm{NIH}=\frac{\Delta \mathrm{fHB}\cdot V\cdot \frac{100-\mathrm{Hct}}{100}}{Q\cdot \Delta T\cdot 1000}\left[\frac{g}{100L}\right]$$

ΔfHb, difference in free plasma hemoglobin at start and end of measurement; V, priming volume; Hct, hematocrit; Q, volume flow; ΔT, time interval.

### 2.5 Statistical analysis

The resulting NIH values are first tested for normal distribution with the Shapiro–Wilk test. A *t*-test is used to compare both species. Hypothesis H_0_ is defined as porcine blood having an equal NIH in the mean compared to the mean NIH of neonatal blood. All analyses used MS Excel (Microsoft Office, Microsoft, Redmond, USA).

Exclusion criteria are defined due to the susceptible process (circuit priming volumes of less than 100 ml). A representative interval of two standard deviations (2σ-interval), representing 95.4% of the measured data in a normal distribution *a priori*, is defined. Results outside the representative interval are excluded from evaluation. In addition, inhomogeneity within the static reference and insufficient mixing also leads to the exclusion of the experiment. Insufficient mixing is estimated when the free plasma hemoglobin (dfHb) decreases during the continuous measurement.

## 3 Results

In total, 10 experiments with neonatal blood were set up, 1 of which was excluded due to priming complications. This resulted in *n* = 9 experiments for the neonatal hemolysis assessment: 9 experiments with porcine blood have been performed, from which 2 were excluded post-experiment due to a non-linear increase of free plasma hemoglobin. This resulted in *n* = 7 experiments being included in the hemolysis assessment. The distribution for neonatal test circuits and a static reference, based on the decision process shown in Supplementary Material 1, are listed in Table 1.

**Table 1:** Distribution of neonatal test circuits.

Number of experiments	Circuits primed	Static reference
1	2	Yes
2	2	Yes
3	2	No
4	1	Yes
5	Excluded	Excluded
6	1	Yes
7	1	Yes
8	1	No
9	1	No
10	1	Yes

**Figure 2:**
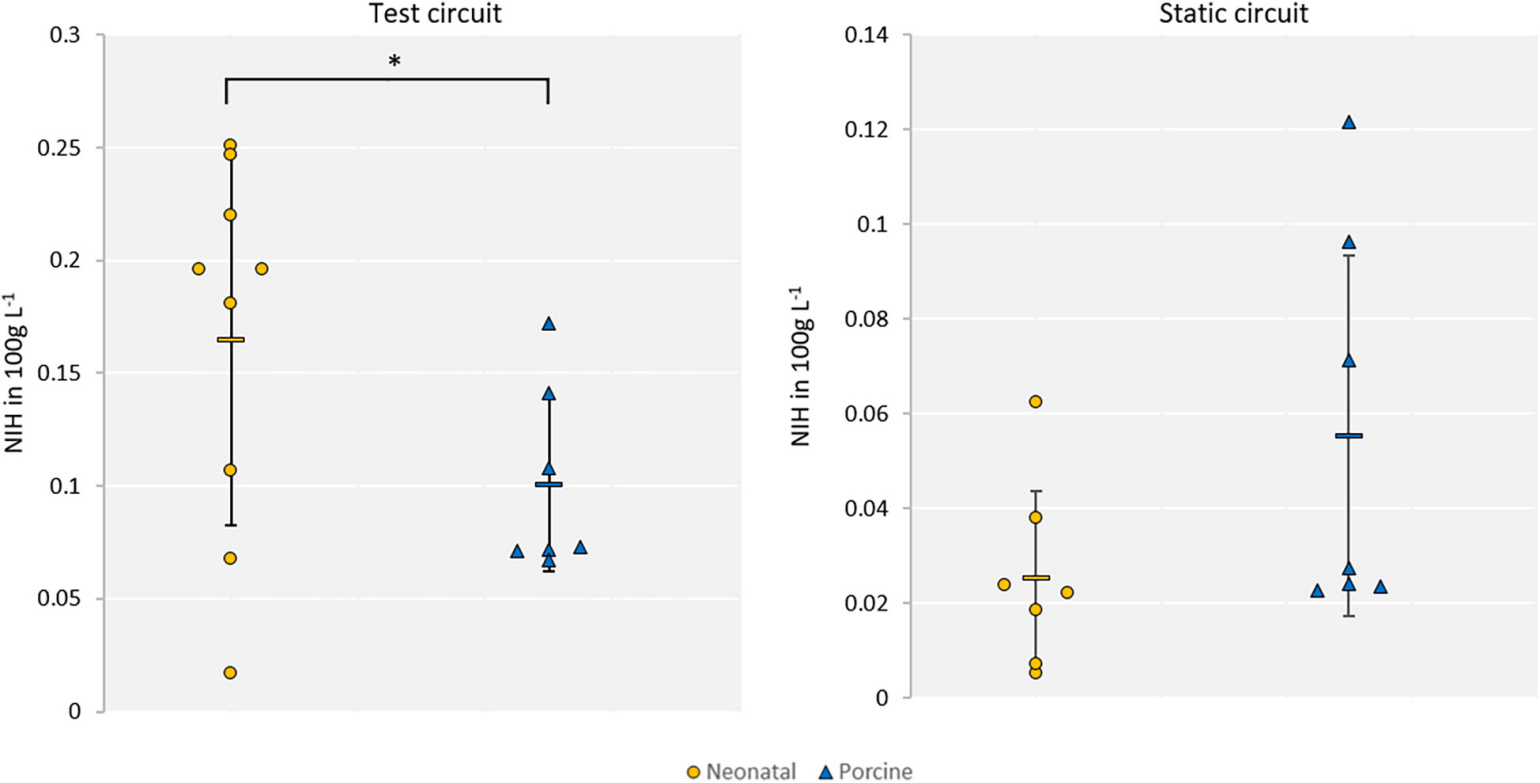
Comparison of neonatal (yellow) and porcine (blue) NIH within the test circuits (left) and static reference (right).

The mean NIH of the neonatal blood was calculated to be 0.165 g 100 L^−1^ (SD 0.082) and 0.025 g 100 L^−1^ (SD 0.018) for the mean neonatal blood static reference. The neonatal results were found to be normally distributed. The porcine blood of nine animals was used in the experiments. Two of the nine experiments were excluded, one due to the non-linear increase of free plasma hemoglobin within the static reference and the other due to insufficient blood mixing within the circuit, resulting in measurement failure at 240 min^−1^. The mean NIH of porcine blood was determined to be 0.101 g 100 L^−1^ (SD 0.038) and 0.055 g 100 L^−1^ (SD 0.038) for the test circuit and static reference, respectively. The porcine results were also found to be normally distributed and are significantly lower (*p* = 0.03) than the neonatal results. NIH results are depicted in Figure 2. The mean delta of the free plasma hemoglobin (dpfHb) values were 33.15 mg dl^−1^ (SD 12.78) for porcine and 54.33 mg dl^−1^ (SD 24.00) for neonatal blood.

## 4 Discussion

The study showed a significant difference in human neonatal vs. porcine hemolysis, as shown by the NIH. Looking at the mean values, a grading score according to Lou et al. (32) would categorize hemolysis as “mild” for our used porcine blood but as “medium” for the neonatal blood within the test circuits. Therefore, an underestimation of hemolysis through neonatal extracorporeal systems may occur when tested *in vitro* using porcine blood. However, the *in vitro* testing hemolysis of adult human blood compared to porcine blood is common practice (15, 16) and is scientifically approved in comparative hemolysis testing.

Based on the significantly different NIH values and standard deviation, neonatal erythrocytes are more susceptible to hemolysis in the tested low-flow, high-pressure operation point. Adult human and porcine blood are in a similar range of hemolysis within the existing models (16). This could explain the increased hemolysis rates in neonatal ECMO compared to adult ECMO reported by ELSO (11, 12) and Dalton et al. (13). Our results suggest that blood-contacting extracorporeal life support devices ought to be designed and tested with particular attention to the higher vulnerability of neonatal erythrocytes.

Concerning the relative hemolysis measurement, the standard deviation of the neonatal NIH is twice as high as that of porcine blood NIH. We assume a higher donor-donor variability regarding hemolytic response within neonates. This results in a lack of accuracy in a realistic comparison between a reference circuit and a test circuit for standard hemolysis testing. Accordingly, simple comparison or conversion factors for the porcine blood results did not suit the aimed patient group scattering. The standard deviations of adult human and porcine blood have been reported to be similar, and standard *in vitro* experiments can be compared regarding their relative hemolytic behavior (14–16). Further studies to prove these assumptions need to be done to define an optimal *in vitro* neonatal blood analog. With a complete investigation of additional *in vitro* animal models, a sufficient decision can be made on an optimal model for human neonatal blood. Furthermore, with an increasing number of experiments, the significant differences in blood composition of the animal models and neonatal blood (18, 22, 23) need to be investigated for their impact on hemolysis.

A higher frailty of neonatal erythrocytes can be assumed due to the lower static reference NIH of neonatal blood compared to porcine and a higher NIH for the neonatal blood within the test circuits. In addition, the standard deviation of the static reference in neonatal blood is markedly lower when compared to the testing circuit, while the porcine standard deviation stays constant. This also indicates a higher frailty of erythrocytes and a higher individuality of blood damage among the donors within the neonatal group. The observed higher frailty can, additionally, be explained by significantly increased deformability (18) and GA dependent differences in viscosity (23) of neonatal human blood compared to adult human blood observed in previous studies. In addition, higher standard deviations of the measured parameters in Linderkamp et al. (18) and Rampling et al. (23) also indicate a higher patient individuality of neonatal blood.

In artificial placenta technology applications, it should be considered that extremely preterm infants qualifying for this treatment are often at risk of acute kidney injury. The ECMO procedure should, therefore, avoid hemolysis altogether. An increased level of plasma-free hemoglobin could lead to additional stress, damage, or failure of the kidneys (13, 33). Hemolysis can also be related to several complications like coagulation, bleeding, and other organ failures during ECMO (13). Adjusting the *in vitro* standard test method could overcome the underestimation of neonatal blood hemolysis. A new analog for neonatal blood must translate dpfHb and NIH values in the correct range, or further investigation of the existing models is needed.

The initial levels of free plasma hemoglobin measured before the start of the experiments and after the mixing and warm-up were valid according to the standards (14). In addition, based on the low static hemolysis of neonatal blood, it can be suggested that the harvesting method for neonatal blood is valid, with low initial hemolysis due to the harvesting process.

The study examined one operational point to investigate the hemolytic behavior of neonatal and porcine blood. The measurement point was an off-label operating point of the DP3 pump to test a worst-case scenario within the low-flow-high-pressure operating range of blood pumps.

## 5 Conclusion

Our experiments revealed a significant difference in hemolysis between human neonatal and porcine blood in a standard *in vitro* hemolysis test. Neonatal human NIH values were found to be significantly higher, with a broader variation, compared to the porcine blood group. The results suggest that standard *in vitro* testing using porcine blood would underestimate hemolysis production in neonatal applications. A translation-oriented animal blood model for neonatal blood needs to be defined.

## Data Availability

The original contributions presented in the study are included in the article/Supplementary Material; further inquiries can be directed to the corresponding author.
