# Supplementary figures and images for: In vitro investigation of elevated hemolysis susceptibility in neonatal blood
Source: Front Pediatr. 2025 Aug 26;13:1616084. doi: 10.3389/fped.2025.1616084 (PMC12417390; doi:10.3389/fped.2025.1616084)

# Supplementary Matrial 1: Decision process for number of circuits

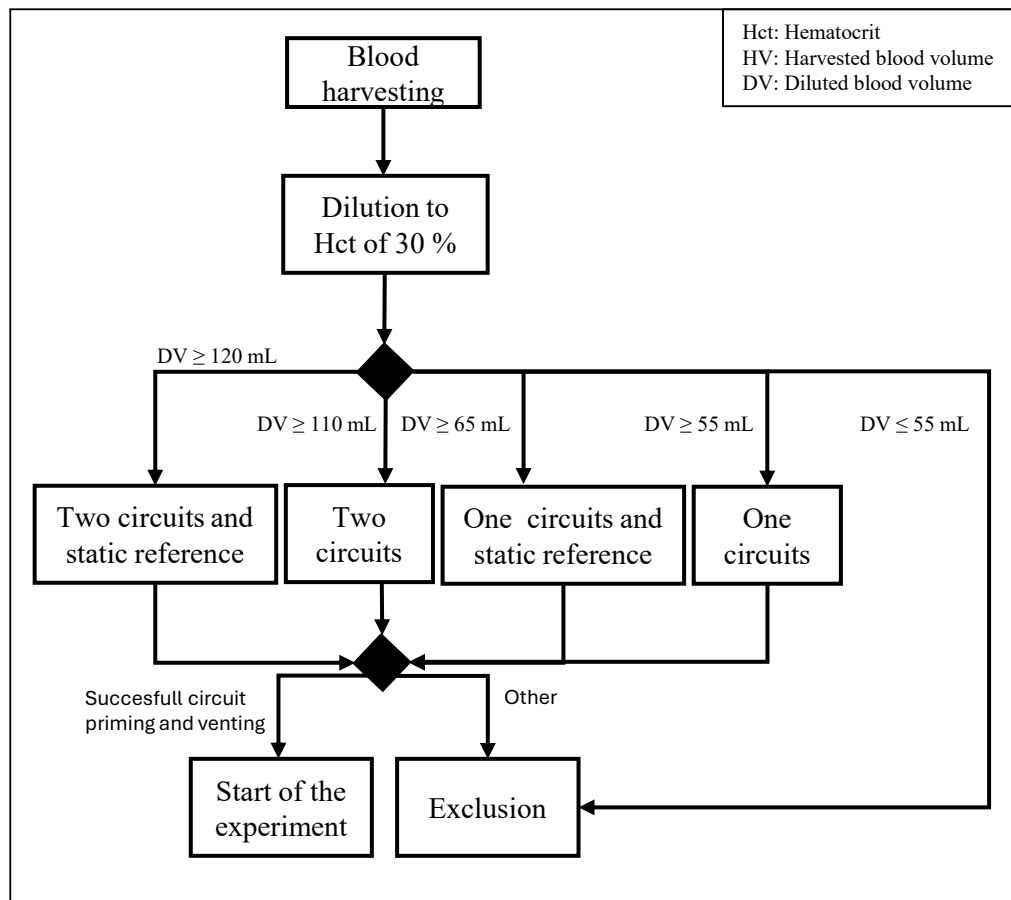

Supplement: Supplementary file 1 [file Supplementaryfile1.pdf]
